# Bipartite Network of Interest (BNOI): Extending Co-Word Network with Interest of Researchers Using Sensor Data and Corresponding Applications as an Example

**DOI:** 10.3390/s21051668

**Published:** 2021-03-01

**Authors:** Zongming Dai, Kai Hu, Jie Xie, Shengyu Shen, Jie Zheng, Huayi Wu, Ya Guo

**Affiliations:** 1 Key Laboratory of Advanced Process Control for Light Industry, Ministry of Education, Jiangnan University, Wuxi 214122, China; 6181913008@stu.jiangnan.edu.cn (Z.D.); xiej2018@jiangnan.edu.cn (J.X.); guoy@jiangnan.edu.cn (Y.G.); 2 Soil and Water Conservation Department, Yangtze River Scientific Research Institute, Wuhan 430010, China; shenshengyu@mail.crsri.cn; 3 The State Key Laboratory of Information Engineering in Surveying, Mapping and Remote Sensing, Wuhan University, Wuhan 430079, China; zhengjie@whu.edu.cn (J.Z.); wuhuayi@whu.edu.cn (H.W.); 4 Department of Bioengineering, University of Missouri, Columbia, MO 65211, USA

**Keywords:** bipartite network, interest, sensors, applications, machine learning, classification

## Abstract

Traditional co-word networks do not discriminate keywords of researcher interest from general keywords. Co-word networks are therefore often too general to provide knowledge if interest to domain experts. Inspired by the recent work that uses an automatic method to identify the questions of interest to researchers like “problems” and “solutions”, we try to answer a similar question “what sensors can be used for what kind of applications”, which is great interest in sensor- related fields. By generalizing the specific questions as “questions of interest”, we built a knowledge network considering researcher interest, called bipartite network of interest (BNOI). Different from a co-word approaches using accurate keywords from a list, BNOI uses classification models to find possible entities of interest. A total of nine feature extraction methods including N-grams, Word2Vec, BERT, etc. were used to extract features to train the classification models, including naïve Bayes (NB), support vector machines (SVM) and logistic regression (LR). In addition, a multi-feature fusion strategy and a voting principle (VP) method are applied to assemble the capability of the features and the classification models. Using the abstract text data of 350 remote sensing articles, features are extracted and the models trained. The experiment results show that after removing the biased words and using the ten-fold cross-validation method, the F-measure of “sensors” and “applications” are 93.2% and 85.5%, respectively. It is thus demonstrated that researcher questions of interest can be better answered by the constructed BNOI based on classification results, comparedwith the traditional co-word network approach.

## 1. Introduction

With the development of information technology, scientific text information has increased dramatically. It is estimated that the growth rate of the new scientific publications is about 9% each year, leading to a doubling of the global scientific output roughly every nine years [1]. Researchers need to explore a domain according to their own questions to identify the specific knowledge information. For researchers interested in cancer diagnosis, the interests could be cancer-related genes and the corresponding proteins [2]. Researchers in the microbiology field may be interested in the microbial habitat and phenotypic information [3]. The information of interest often appears as knowledge pairs, which are also commonly seen in many real-world scenarios, like the pairs of readers and books, actors and movies, audiences and music. These pairs can be effectively represented by a bipartite network, the patterns of which can be computed in a shorter time than that of a general complex network. Therefore, a bipartite network can used as an important type of network for pattern extraction, further data mining and visualization. This network is a special type of the complex network, with only two sets of specific nodes. In our case, the specific knowledge pairs like “genes and proteins”, “microbial habitat and phenotypic information”, and other pairs can be well modeled and visualized as the two sets of nodes in a bipartite network. Thus, the overwhelming amount of information can be reduced and the specific knowledge information can be highlighted.

However, in bibliometric or scientometric theory, co-word networks [4] or co-citation networks [5] are more often seen. These networks provide the theory foundations for representing and analyzing the knowledge landscape. They can be regarded as a general type of complex network, which is less effective for computing or finding patterns than a bipartite network. Transforming the complex network into a bipartite network may provide a way to effectively explore the knowledge of interest. This is because co-word networks are built based on the keyword list for each of the articles. Some of the keywords are even suggested by the rough keyword classification tree of the journal submission systems. Therefore, detailed information about the conducted research may be missing. In addition, the chaotic terms in the co-word network may confuse researchers, because of the inclusion of very different types of words like real-world datasets, technical notions, academic concepts, or names of hardware. Moreover, the results may not be ideal due to a lack of consideration of the preferences or tendencies of the researchers in a specific field. Co-word networks often provide researchers with over-general concepts. This could be a waste of time for an experienced expert.

Experienced researchers often want to know more specific information, like the pairs of entities. Taking the “remote sensing” field as an example, an important question for “remote sensing” researchers may be “what sensors have been studied in what applications” since studies in the remote sensing field are often highly related to the sensors used. Sensors determine the resolution, the spatial-temporal precision, and other attributes of the acquired data. High-resolution sensors can provide urban planners with background images, helping road network construction. Data acquired by night-time light sensors can help evaluate human economical activities [6]. Moreover, data from several different sensors can sometimes be used simultaneously. High-spatial-resolution sensed data can cover the shortage of the high-temporal-resolution but low-spatial-resolution data [7]. Two sensed data can be combined to generate new information [8]. Co-word networks from traditional scientometric methodology can offer a macro view of the remote sensing-related field [9] and other scientific fields [10,11,12]. However, co-words are often based on keywords which are often too general without enough details. How to extract the specific and concrete pairs of “sensors” and “applications” thus becomes an open question.

Mining the valuable literature to generate very specific and concrete knowledge pairs is not a new topic. Scholars have treated this topic as a serious science and put forward the corresponding theory, namely the theory of the solution of inventive problems or TRIZ [13]. Several very useful principles for innovation have been proposed by manually organizing and analyzing the high-quality patent and literature like 50 years ago [14], but nowadays, this work may be able to be reproduced by the modern text mining technologies. Methods of text mining nowadays can be roughly grouped into four categories: statistical approaches, linguistic approaches, machine learning approaches, and other approaches [15]. Some studies have applied the text mining techniques to model more abundant and accurate semantic meanings [16]. Several feature extraction methods from text mining have been used to identify problems and solutions [17] or more representative domain keywords [18]. Using text mining, it is possible to extract knowledge pairs, further building the bipartite network. As the knowledge pairs reflect the specific interests of researchers, we call this network consisting of knowledge pairs the bipartite network of interest (BNOI).

As we are familiar with the field of the remote sensing, we build the BNOI using the literature from the remote sensing field, answering the typical question “what sensors can be used for what applications”. With manual annotation, we determine whether a sentence belongs to the category “sensors” or not and we also determine whether a sentence belongs to the “application” type or not. These sentences with annotations will be used to build the training datasets and the testing datasets. If a “sensors”-containing sentence and sentence with the word “application” appear in a same paper, then the bipartite network relation can be built. When the words “sensors” and “applications” are visualized in a bipartite network, the experts can get more concrete information for questions of interest rather than the over-general information available in a co-word network. Thus, a lot of time and energy of the experts will be saved. Consider the following examples:

- “Both the clumping index (CI) and leaf area index (LAI) can be obtained from global Earth Observation data from sensors such as the Moderate Resolution Imaging Spectrometer (MODIS).” [19]
- “The Operational Land Imager (OLI) onboard Landsat-8 satellite can provide remote sensing reflectance (R-rs) of aquatic environments with high spatial resolution (30 m), allowing for benthic habitat mapping and monitoring of bathymetry and water column optical properties.” [20]

These two example sentences are identified as both “sensors” type and “applications” type. From the first sentence, we know that the Moderate Resolution Imaging Spectrometer (MODIS) can observe the clumping index (CI) and leaf area index (LAI). From the second sentence, we know that Landsat-8 satellite can map the benthic habitat and monitor the bathymetry and the water column optical properties. However, this is concluded by human readers through reading and interpretation. To automatically understand the linkages of “sensors” and “applications”, text mining techniques will help. Nine feature extraction methods (traditional models and neural network language models (NNLM) [21]) and three classical classifiers (SVM, NB, and LR) are employed. By comparing different feature combination and classification assembly methods, classification models are obtained. 

To be more clear, feature-level fusion (fusing nine features) and the best classifiers identification are adopted in our paper, which has been also adopted in reference [17], but differently from the former work, in order to increase the classification performance, the concept of multi-attribute group decision-making is borrowed by using the voting principle (VP) [22,23]. After identifying the classifiers with the best performance the VP method is used for the classifier-level fusion. “Sensors” and “applications” are then automatically identified through the classification process. Based on the best classification results, the BNOI for “sensors” and “applications” is built and compared with the traditional co-word network results. 

The contribution of our research is to fill the gap of not considering the interests of researchers in traditional scientometric analysis. The approach can be used to enhance the visual and analytical scientometric software like VoSviewer [24] or CiteSpace [25]. Armed with the interests and domain preferences of researchers, the visualization of the domain knowledge can become more of interest to experienced domain experts. The following sections are organized as follows: how the work related to our task is done is described in Section 2. Section 3 describes the methodology and the workflow that we will use for experiments, including experimental design, classification techniques, and corpus creation. Section 4 provides our experimental results and analyses. Section 5 discusses the results and draws the conclusions.

## 2. Related Work

Mining the patents and literature to help find the rules of innovation thus helping better innovation process in the scientific field is an important topic [13]. TRIZ, known as the theory of the solution of inventive problems, has been developed by analyzing over two million references and patent files in a early period [14] without advanced text mining techniques, and many useful invention principles were proposed. Nowadays, the innovation happens more frequently and a larger amount of patents and literature are available for mining. Therefore, mining the literature for concrete innovations with proper text mining techniques become a new challenge.

Text mining is greatly developed under the urgent demands of mining of open-domain text nowadays [26]. Online social media platforms such as Facebook, Twitter, and Weibo generate a large amount of text contianing public opinion data every day, and machine learning models can be used to analyze these data to understand the social phenomena of public concerns [27]. Text mining is also used to mine quality information for helping extract useful knowledge [28], sentiment classification for film reviews [29], and multilingual sentiment analysis [30]. However, these methods all deal with the text from the open domain, which is a different problem than text processing in scientific papers that are often in a very specific domain because, scientific text from scientific papers is often more standardized, having very professional vocabularies, and being more objective from the emotion perspective.

Therefore, the famous text mining methods cannot be directly applied for this mining task, or at least, the text mining methods should be adjusted thus better serving the mining task in scientific texts. For example, for open domain, extracting the knowledge pairs can be undertaken by the name entity recognition (NER) task and the entities are often very concrete objects like people, locations and dates [31]. However, such entity types are usually not sufficient for the machine to understand the facts contained in a scientific text, which often contain more abstract and complex meanings. 

Currently, these text mining methods have been selected or adapted accordingly for dealing mining task of scientific texts. Several approaches have used text mining methods to classify articles, whereby classifiers are adjusted or features selected for fitting the classifying scenario. For example, neural networks and SVM are adjusted for classifying publications from the fields of life science, resource and environment science, and basic science [32]. Besides classifiers, features are also vital for classification performance. Several traditional features are extended or adapted for analyzing literature text. For example, the methods considering the weights of words for documents are applied to divide scientific documents into art, biology, literature, and so on [33,34]. Other traditional methods such as the bag of words (BOW) and term frequency (TF) are also frequently applied. For example, by using supervised machine learning to classify text based on TF [35], documents are classified using improved term frequency–inverse document frequency (TF-IDF) results [18,36].

These adaptations provide meaningful insights for us to automatically understand scientific papers. Recently, a work classified the knowledge pair of “problems” and “solutions” from scientific research papers; Different linguistic features and NNLM features were tested for finding the best combinations [17]. Their work offered an insight for exploring the interests of researchers. “Problems” and “solutions” are apparently the most frequently mentioned knowledge pairs. Similar to the TRIZ theory [13,14], the problems and solutions are the key research targets in their work. Differently, their work provides a practical solution for how modern text mining technologies can be used for extracting the interested knowledge pairs, so this work can be regarded as the modern approximation version of the TRIZ process, providing intelligent suggestions for problems and solutions. 

In addition, there is still space for improving the classification accuracy, because only single classifiers are used in that work [17]. A combination of these classifiers using the voting principle (VP) may be a direct solution for improving the accuracy, which has been mentioned by many previous works. For example, Duan et al. proposed an extreme learning machine based on voting principles to determine the hidden layer in the neural network and reduce randomness [37]. Hull et al. considered the simple probability average strategy of four text filtering methods [38]. It is found that this strategy can improve the best classifier for sorting documents and is always better than the best single algorithm in filtering applications, but Li and Jaen found that a simple average combination of multiple classifiers does not always improve classification accuracy compared to the best single classifier [39]. Uren pointed out that voting systems with different features will perform better than voting systems with a single feature [40]. Therefore, VP methods could be promising in the classification task.

Inspired by these works, in our paper, we proposed to mine the knowledge of interest pair of “sensors” and “applications”. Our work can be regarded as an extensional work of [17] because we also use a similar solution, with traditional and NNLM feature extraction methods to identify the terms “sensors” and “applications”. We aim at a different knowledge pair of “sensors” and “applications”, which can be representative for a large type of data-dependent research activities. With the method adapted from [17] and the new introduced NNLM features like FastText, ELMo, and Bert, the classification results are generated. Finally, based on the classification results, information of interest visualization of BNOI is provided for researchers. 

## 3. Materials and Methods

### 3.1. The Complete Workflow 

Our new corpus is constructed based on 350 articles in the “remote sensing” field from the Web of Science core collection database in 2017 and 2018, the same database used in many scientometric analysis [9,12]. We used the following search constraints:

*TOPIC: (“remote sensing”)*

*Refined by: DOCUMENT TYPES: (ARTICLE).*
*Timespan: 2017–2018. Indexes: SCI-EXPANDED.*

**Figure 1:**
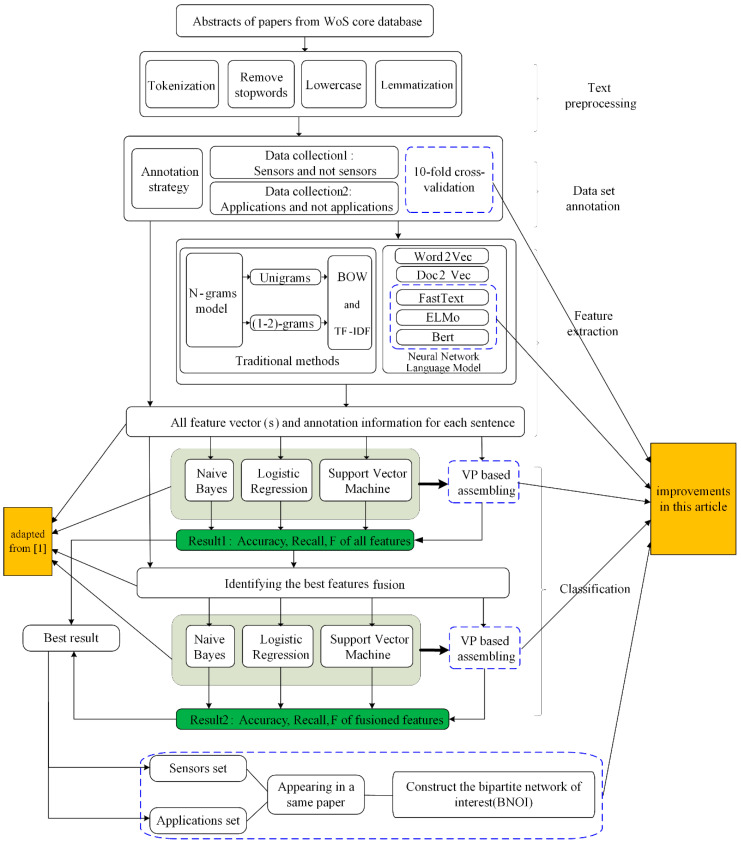
“Sensors” and “applications” classification and BNOI construction (result 1 and 2 will be compared and evaluated).

We extracted the abstract section, then split the abstracts into sentences. Figure 1 shows the general process of the entire system. In this work, 350 abstracts are divided into 3317 sentences. We manually label these sentences to determine if they belong to the “sensors”, “applications”, or others. 

*Definition for whether a sentence or a key phrase is belonging to “sensors”:* an expression containing technique details or the short name of a certain sensor product will be considered as belonging to “sensors”. Example expressions include “Synthetic aperture radar (SAR)”, “Moderate resolution imaging spectrometer (MODIS)”, etc.

*Definition for whether a sentence or a key phrase is belonging to “applications”:* an expression containing the concrete actions for certain purpose will be considered as belonging to “applications”. Key action words include for example observation”,” mapping”,” characterization”, “identification”, etc.

Based on these definitions, we asked three researchers with a remote sensing background to perform the annotation. Every sentence containing concrete names of sensors will be considered as belonging to the type “sensor”, otherwise it will be considered as “non-sensor”type. Similarly, sentences containing concrete applications will be considered as belonging to the “application” type, otherwise they be considered as the “non-application” type. Two researchers were asked to annotate the same sentence, and if the tags do not match, the third researcher will use the Word2Vec method to obtain the semantically similar words (Table 1 and Table 2) of the target words “sensor” and “application” to determine which type the sentence belongs to.

After the annotation process, we start to find the best combination of the classification methods. Many text classification methods like traditional method (N-gram models, TF-IDF, and BoW models) and the developmental versions of NNLMs [21] (like Word2Vec, Doc2Vec, FastText, ELMo, and Bert) can be used for this task. In this paper, only the word representations in the word vectors form are used. However, these methods normally deal with text from the open domain, which is different from the text processing in scientific papers because scientific text from scientific papers is often more standardized, having very professional vocabularies, and being more objective from the emotion perspective. Moreover, the terms in the scientific text are often more general and have complex meaning rather than the simple semantics for words used in open domains. Therefore, we use aworkflow to help select the best combination for the classification task. For the workflow we also refer to the work [17].

After a series of pre-processing steps of these sentences, we prepared to use the 10-fold cross-validation method for experimental verification. Therefore, the datasets are split into ten sets of training and test data collections for the later classification process. Then, we used a total of nine feature extraction methods (both traditional and NNLM) to obtain features. These feature methods are described in detail in Section 4, including traditional methods like BOW, TF-IDF, and NNLM methods like Word2Vec, BERT. After the word vectors are generated, we choose to use three classifiers including NB, SVM, and LR. We check the effects of a simpler method of classification on the final outcome first before investing heavily in their implementation. In addition, we also use an ensemble method, voting principle (VP), to help enhance the performance of the classifiers. This method combines the decisions of multiple algorithms, so a more robust classification model with higher accuracy can be built [41]. Meanwhile, based on the classification results, we also select features with good performance for feature fusion and use them as input features. 

### 3.2. Training Data and Testing Data Setup

We use supervised classification methods to build BNOI to identify whether the sentences belong to “sensors” or “applications”. To simplify the task, we only consider classifying sentences and we examine the dependencies and look for the target word (“sensors”/”applications”) in the subject location and select them as candidate corpus words or phrases. In order to find as many wording different descriptions of sensors and applications as possible, we additionally use semantically similar words (nearly synonyms) of the target word “sensors” and “applications” to search. Cosine similarity was used as the semantic similarity measuring method in a distributional vector space, and Word2Vec [42] is used to train the data set. Word2Vec can not only effectively capture the semantic similarity of words from a huge text corpus, but also the syntactic similarity [43]. From the 200 words which were semantically closest to “sensors” and “applications” we exclude meaningless and erroneous words. Table 1 and Table 2 show some of them. These phrases also served as an auxiliary role in the labeling process of the three researchers. These tagged datasets are also available in the project on the GitHub (https://github.com/15514783351/RemoteSensing. Available from 30 October 2020). The abstracts are sliced into sentence collections. The tags of “whether belonging to sensors” or “whether belong to applications” are sequence of “0” and “1” in the corresponding tagging files. “0” and “1” in sensor tagging file stands for the sentence does not belong to “sensor” and belongs to “sensor”, respectively. 

For ease of expression the phrases are represented in Table 1 by the prototype of the word. For example, the hyperspectral and multispectral belong to the category ‘spectrum’. It can be concluded from Table 1 that there are mainly some special remote sensing satellites, long wave, short wave, and infrared related to sensors.

Unlike remote sensing, applications often do not have a particularly obvious style, and classification usually requires consideration of multiple words. In the manual annotation process, there is no doubt that words with such as “application” and “task” are often positive samples. Since the classification object is a sentence type, the application may be included in the context. Therefore, it is assumed that two of the three elements are included as a positive sample according to the marking strategy. These three elements are application subject (mainly sensors), application content (monitoring or mapping, etc.), application object (vegetation, ocean), respectively. Some application-related words are listed in Table 2.

### 3.3. Feature Extraction, Feature Fusion, and Classical Classifiers

#### 3.3.1. Features Extractions

After the corpus creation, the feature extraction is the key process of the further classification. The feature extraction is to convert unstructured data such as text into structured data. This is also the difficulty of the experiment. When we get the structured data, we can use it in more machine learning algorithms, even deep learning algorithms, such as convolutional neural networks (CNN), recurrent neural network (RNN), and so on. For feature extraction, there are traditional methods (N-grams) and NNLM methods (Word2Vec, FastText, BERT, etc.).

Traditionally, the unigrams (1-g) model has been successfully used for classification tasks in NLP [44]. In the data set, there are more than one word or even more words representing remote sensing, such as “thermal infrared” and “moderate resolution imaging spectrometer” in Table 1. However, when the feature is only the “sensors”, the label is annotated as negative. This is because the feature may not be a sensor in the remote sensing field, which will cause ambiguity. Therefore, in order to better represent adjacent word relationships, we also used (1-2)-grams. In practical applications, N ≤ 3 is usually selected [45]. As N increases, the dimensions will become so large that even a ‘dimensional explosion’ will occur. For n-grams, there are usually two representation methods, the BOW model and the TF-IDF model. BOW is a common method used for building a vector representation of text documents [46]. The key of this approach is that the text sentences can be expressed using unordered set of frequencies of selected words (or dictionary) [47]. The TF-IDF based on the BOW scheme in which a document also can be represented by a collection of words used in the document [48]. However, TF-IDF is slightly different from BOW. The former not only needs to consider the frequency of words (or dictionaries) in the document, but also the number of times the words (or dictionaries) appear in the corpus. From this, the relative importance of the words (or dictionary) is inferred and the sentence is vectored (by adding word vectors). Although the N-grams model was proposed long time ago, it still has good performance in many NLP scenarios. However, N-grams mainly focus on information such as word frequency, and pay less attention to hidden information between words. For the classification of “applications”, the feature information is often distributed in large range in the longer sentences. For example, remote sensing can obtain “clumping index (CI) and leaf area index (LAI)” [19]. Traditional feature methods may not meet the requirements, so this article also uses NNLM methods for feature extraction.

Different from the traditional methods, the NNLM methods use the language neural network to model the semantics among the words, several classical methods are used, including Word2Vec, FastText, ELMo and Bert. Word2Vec is a tool that Google introduced in 2013 to train word vectors. It provides a way to represent text using distributed vectors. Word2Vec model different from N-grams and TF-IDF, it uses a method of embedding. Word2Vec pays more attention to the context of words. It converts high-dimensional discrete data into low-dimensional dense data. Its dimensions are usually 100–300. The relevance of the context is considered, and two models, skip-gram and CBOW (continuous bag of words) are proposed and implemented in Word2Vec model. Skip-gram predicts the context around it through intermediate words; instead, CBOW uses context to predict the words in between. In CBOW a word is used as the output and its context as input, while in the Skip-gram model it is done the other way around. After that, Google proposed the Doc2Vec model again in 2014 [49], and Facebook proposed the FastText model [50]. Their basic ideas are similar to Word2Vec. The improvement of Doc2Vec over Word2Vec is the addition of paragraph vectors, and FastText adds N-grams model inside words.

Unlike the Word2Vec or FastText, embedding from language models (ELMo) represents the entire input sentence. It uses a two-layer bidirectional language model (biLM) with large-scale pre-training based on character convolution to generate word embedding. The ELMo model is often the sequence model, and its effects have been verified on the corresponding NLP tasks [51]. The word embedding includes grammatical semantics and environmental features. Later, a transformer structure composed of encode and decode are proposed, outperform many models like CNN or RNN in text classification [52]. Bidirectional encoder representations from transformers (BERT) is based on the transformer concept and uses a multi-layer bidirectional structure [53]. At each layer, it iteratively revises the representation of every position by exchanging information across all positions at the previous layer in parallel with the transformer layer. Based on this mechanism, BERT has the ability to directly capture semantic dependencies of large distance, so it is also been used as one of the feature extraction method. The model has been pre-trained on a large corpus and related model parameters are provided:

#### BERT-Base, Uncased: 12-Layer, 768-Hidden, 12-Heads, 110 M Parameters

The model will generate a total of 12 layers of word vectors. In this experiment, the last layer is used as the experimental data. Each of the models have their advantages in the NLP tasks, but they are used for the open-domain text mining and we do not know if they can work well on our scientific text mining task. Thus, we have selected a total of nine feature extraction methods, each of the mentioned models (unigrams (BOW), unigrams (TF-IDF), (1-2)-grams (BOW), (1-2)-grams (TF-IDF), Word2Vec, Doc2Vec, FastText, ELMo, Bert) acting as one feature extraction method. Note the first four traditional methods have not used the pretrained models and all the NNLM-based model have used the pretrained models.

#### 3.3.2. Classical Classifiers

Many novel text classification algorithms have been proposed in recent years. The three classical classifiers (NB, SVM, and LR) used in the paper are described. The NB model is one of the most basic statistical classification methods. The model has been widely used as a probabilistic learning model for text classification [54,55,56]. The SVM as another classifier, has been successfully applied in text classification [57]. The core idea of SVM is that in high-dimensional space, finding an optimal hyperplane that separates the positive training samples from the negative one [58,59]. In many scenarios, SVM can achieve good results when data amount is small. The LR is another commonly used classifier [60,61,62]. Typically, the LR model is a member probability that computes one of two categories in a dataset. The posterior probability of the output can facilitate the classification result. These three classical classifiers have been used as the benchmark for the testing the proposed voted principle method, which combine the ability of these ability of the classifiers like ensemble learning. For these three classifiers, the Scikit-learning machine learning library provides the implementation [63].

#### 3.3.3. Vote Principle

**Figure 2:**
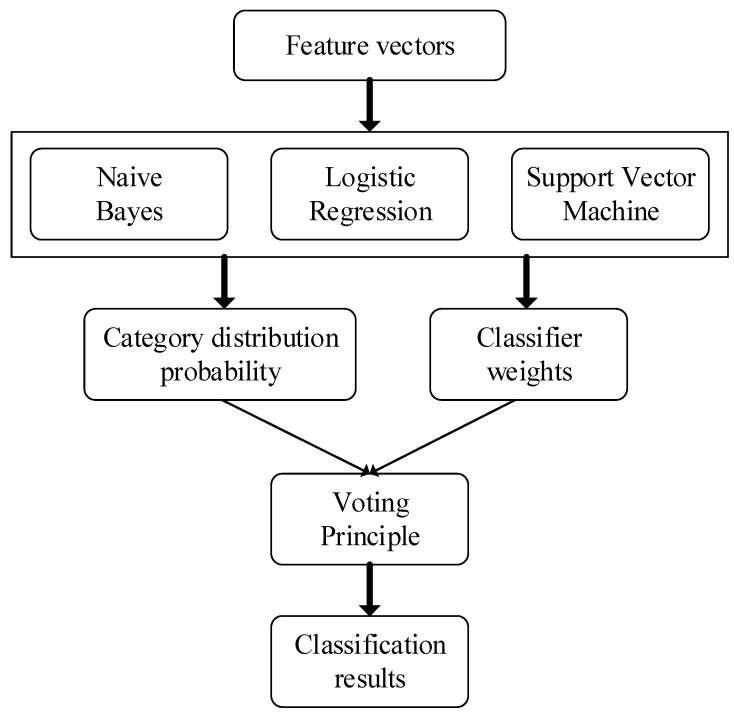
Voting principle.

In addition, to improve performance, we also use the VP method similar to ensemble learning in this experiment. The main idea is shown in Figure 2.

In Figure 2, the classifier weights of NB, LR and SVM are determined according to the F-measure of the positive category of the training sample classification result. The specific method is shown in Equations (1) and (2). The categories distribution probability is decided by the prediction results of the three classifiers for the test samples:

(1) $$F^{*}\left(y_{i}\right)=\{\begin{array}{ll} F\left(y_{i}\right)+F\left(y_{i}\right)\times 10\%, & if\;F\left(y_{i}\right)\;is\;best\\ F\left(y_{i}\right)-F\left(y_{i}\right)\times 10\%, & if\;F\left(y_{i}\right)\;is\;worst\\ F\left(y_{i}\right), & other \end{array}$$

(2) $$W\left(y_{i}\right)=\frac{F^{*}\left(y_{i}\right)}{\sum_{k=1}^{3}F^{*}\left(y_{k}\right)}\;i=1,2,3$$

where $y_{i}$ is the classifier (NB, LR, and SVM), $F\left(y_{i}\right)$ is the F-measure of $y_{i}$, and $W\left(y_{i}\right)$ is the weight of $y_{i}$. The voting principle will combine the weights of the three classifiers and the probability of test sample category distribution to determine the final result, as shown in Equations (3) and (4):

(3) $$P_{{}_{i}}^{*}=\sum_{k=1}^{3}W\left(y_{k}\right)\cdot P{\left(y_{k}\right)}_{i}\;i=1,2,\cdots ,n$$

(4) $$VP_{i}=\{\begin{array}{ll} 1, & P_{{}_{i}}^{*}\geq 0.5\\ 0, & P_{{}_{i}}^{*}<0.5 \end{array}$$

where $P{\left(y_{k}\right)}_{i}$ represents the probability that the classifier $y_{k}$ identifies the positive class for sample i. For example, if the F-measures of {LR, NB, SVM} are {80%, 70%, 50%}, the weights are {0.433, 0.345, 0.222} according to Equations (1) and (2). There is a sample X, the output probabilities of the three classifiers are {60.0%, 50.0%, 30.0%}, and the VP output probability is 49.89% by Equation (3). The final classification result of this sample is negative class (0) by Equation (4).

From Figure 2 and Equations (1)–(4), it is easy to conclude that the voting results in the three classifier results are selected. For the VP classifier, the classification result will be improved and the model will be more robust. For classification tasks, three evaluation indexes of precision, recall, and F-measure are usually used. The existing category C, the confusion matrix of the file classification is shown in Table 3.

The precision, recall, and F-measure of the category C are shown as follows:

(5) $$precision=\frac{TP}{TP+FP}$$

(6) $$recall=\frac{TP}{TP+FN}$$

(7) $$F-measure=\frac{2\times precision\times recall}{precision+recall}$$

These three evaluation indexes will be used as the performance indicators for the final estimation of the selected combination of best features and classifiers.

#### 3.3.4. BNOI Construction

**Figure 3:**
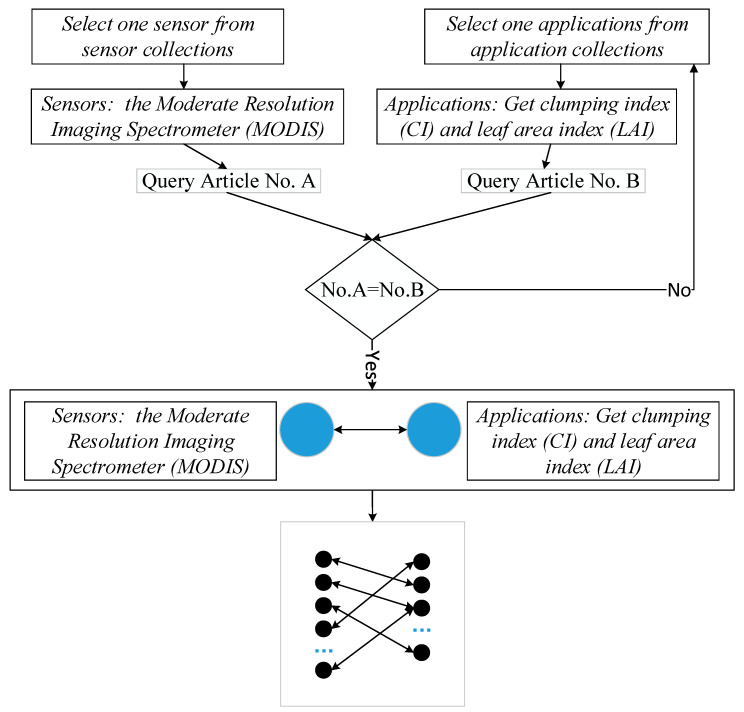
BNOI construction based on the classification results.

The results of the classification are two data collections, one for “sensors”, and the other for “applications”. Note some of them have overlapping sentences. Then, with the classification results, the interested information of “sensors” and “applications” is extracted from the text. Similar to the co-word network construction, the BNOI is also built based on the co-existent relationship between the terms. Figure 3 shows the basic workflow of constructing a BNOI of “sensors” and “applications”.

The BNOI construction process is similar to the construction of a co-word network. If an entity of one type appears in a same paper with another type of entity, they will be connected in the bipartite network. As shown in Figure 3, MODIS can be used to compute the clumping index (CI) and leaf area index (LAI). Therefore, MODIS as a sensor will be connected to the application of “get CI and LAI”. As the amount of papers accumulates, the nodes and the edges of the bipartite network will grow, so, the BNOI can be regarded as a co-word network after automatically filtering irrelevant general terms, and only knowledge pairs of interest to researchers will be preserved.

## 4. Results

### 4.1. Middle Results of Text Preprocessing

Text preprocessing usually includes tokenization, case conversion, removing stop words. In addition, the number of selected features (words) can be often reduced by transforming the words to their generic form (lemmatization) [64]. The lemmatization is to restore a formal vocabulary of any kind to a general form (expresses complete semantics), for example, ‘ate’ can be restored ‘eat’. The semantic information of some words will be lost after lemmatization. For this operation, in the “sensors” experiment, we compared the impact of unigrams (BOW) and Word2Vec’s classification accuracy as shown in Table 4.

It can be seen from Table 4 that lemmatization and non-lemmatization changes have little effect on classification accuracy. In methods 1, and 2, the NB and SVM classifiers are less effective, and the LR classifier achieved the highest F-measure of 85.9%. For methods 3 and 4, the accuracy of non-lemmatization is 0.6% higher than that of lemmatization. On the other hand, from the two sets of comparative experiments, it is found that the recall rate of lemmatization is usually higher than that of non-lemmatization. This means that lemmatization allows more sentences to be recognized as positive samples, which will inevitably lead to a decrease in precision. However, the purpose of this article is to provide researchers with as much useful information as possible. In summary, lemmatization does lose part of the semantic information and reduce the accuracy rate. Even so, it is acceptable to adopt lemmatization in this experiment, because this method can not only reduce resource consumption, but also provide more positive sample.

However, using only the feature extraction method of lemmatization, the number of features is still huge (≈5000). A huge number of features will decrease the computing efficiency. Feature reduction is usually the next work during the feature extraction engineering. In this article, we use chi-square statistic (CHI) to select features to reduce the complexity. Table 5 shows the comparison of the number of features using feature selection for BOW feature extraction methods.

From Table 5, after feature selection, the number of features is greatly reduced, and the reduction rate reaches more than 80%. For the “sSensors” or not “sensors” task, the number of features is changed from 5293 to 879, and for the “applications” or not “applications” task, the number of features is changed from 5293 to 970. Redundant features are greatly reduced, and program execution efficiency can be further improved.

### 4.2. Classification Results and BNOI Network Building

The classification is conducted for determining whether a sentence belongs to “sensors” type or not, “applications” type or not. For sensors, the terms of sensors are often just composed of nouns, but for applications, the determination of positive samples often depends on a relatively complete verb-object construct. We used the Stanford CoreNLP analysis tool to obtain related phrases about “sensors” and “applications”. After summary of the statistics, there are about 2269 “sensors”. There are about 4181 “applications”. For the data set, there are 0.67 “sensors” and 1.26 “applications” per sentence.

#### 4.2.1. “Sensors” or Not “Sensors” Classification

As seen from Table 6 and Table 7, we were able to achieve good results in distinguishing whether a single sentence belongs to the “sensors” class in remote sensing. The VP classifier achieved quite good results in the nine feature extraction methods, six of which had the highest F-measure. The main reason is that the VP classifier can fuse information from multiple “decision makers” (classifiers). For example: “*Ground-based radiometers appeared to be highly sensitive to F/T conditions of the very surface of the soil and indicated normalized polarization index (NPR) values that were below the defined freezing values during the morning sampling period on all sampling dates*” [65], obviously, the sentence belongs to the field of remote sensing. The classifiers {LR, NB, SVM} have the probabilities of classifying a positive sample as {30%, 99%, 32%}, and the F-measures are {86%, 82%, 89%}. Calculated by Equations (1) and (2), the weights are {0.3342, 0.2863, 0.3795} respectively. The final result of the VP classifier is 0.501, which is a positive sample. In the traditional feature extraction method (No. 1–4), the (1-2)-grams (BOW) achieves a good performance of 92.9% for the VP classifier. In the NNLM method (No. 5–9), the ELMo method achieves the highest F-measure in the SVM classifier, which is 85.9%.

Since the corpus used in this work is an ultra-short text of a single sentence, the available features are relatively few. For the determination of whether it belongs to the sensor in remote sensing, the useful feature words are mostly single nouns (such as the name of the Remote Sensing satellite) and these words are neutral and not emotional. Using a traditional BOW model may have a good effect. Table 8 shows the chi-square statistic score and P_value for the top lemmas from the unigrams (BOW) in previous binary classification of “sensors” and “non-sensors”.

For the chi-square statistic, the higher the score, the smaller the *p*-value, the higher correlation of the feature will be [66,67]. Table 5 shows the top 10 highly correlation words we selected. The terms “Resolution”, “Image” and “MODIS”, all belong to “Moderate resolution imaging spectrometer (MODIS)”. The next few words are related to spectral imagers. The terms “Landsat” and “Radar” also play a very important role in the classification results. “Landsat” and “Sentinel” are important satellites in the remote sensing field. The results show that these feature words have enough information to build the classification model, and also indicates that the corpus we created is valid. However, it was found that the scores of “remote” and “sense” were too high, which may bias the classification. Therefore, we deleted similar words and the classification results are shown in Table 9 and Table 10.

**Figure 4:**
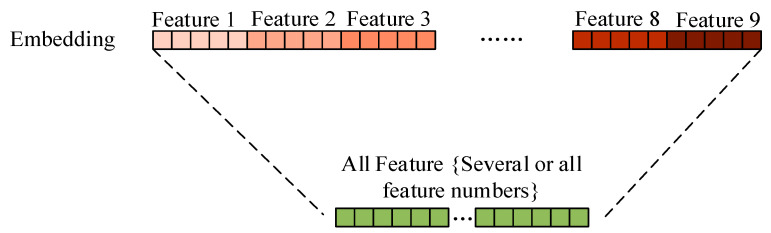
Combination of several or all features.

From the comparison of Table 9 and Table 10, and Table 6 and Table 7, it can be concluded that by deleting some words that cause bias, the classification F-measure of the first nine methods is reduced to some degrees, from 92.9% (Method 3, VP) decreased to 89.3% (Method 3, VP). In this experiment, many language models are introduced, but these models are often used for open-domain text modeling, therefore, these models cannot guarantee the models can still work well in the scientific text scenario. Also, we are also inspired by the work of problem and solution identification [17]. Thus, we used the method of combining multiple features. Its purpose is to fuse word frequency information and semantic information, which can improve each single feature, thus enhancing the classification result. The methods 10 and 11 of Table 6, Table 7, Table 9 and Table 10 use the method, as shown in Figure 4. For the method 10, all the feature methods were combined. The F-measure is higher than all single methods. For the method 11, several high-precision feature methods {1, 3, 7, 8} have been selected, and the final classification F-measure is the highest, which is 94.0% (Table 7) and 93.2% (Table 10).

For the method 11 in Table 10, the confusion matrix of VP classifier is shown in Table 11. For sensor classification, the precision, recall and F-measure are 93.1%, 93.3%, and 93.2%, respectively. From the above three evaluation indicators, we can tell the VP classifier can improve the performance of the sensor classification.

#### 4.2.2. “Applications” or Not “Applications” Classification

The results for disambiguation of applications from non-applications can be seen in Table 12 and Table 13. Among the first nine methods, as well as the classification results of “applications” in remote sensing, the SVM classifier achieved the best results, and the five feature methods had the highest F-measure.

By analyzing Table 12 and Table 13, when combining features (method 10 and method 11), the VP achieved the best classification result of 86.4%. This shows that the combined features and VP classifier are effective for “applications”. Unlike sensors, application features are more in the form of phrases, so (1-2)-grams (BOW) are used to extract the lemma. Table 14 shows these lemmas.

We selected the top ten highly correlation lemmas. The representative keyword for “applications” are expected to be “monitoring” and “observation”. However, there are also sensors in remote sensing, such as MODIS, Lidar. This is because application is often expressed as the scenarios with concrete actions. Similarly, we also delete biased words, and the classification results obtained are shown in the Table 15 and Table 16 below.

In comparison, after deleting bias words that may cause prejudice, the classification result also appears to be slightly reduced. For method 10, the classification F-measures of combining all features ranges from 84.9% (VP, Table 13) to 84.5% (VP, Table 16). For the method 11, four feature methods {1, 3, 6, 9} were combined and the classification F-measures is reduced from 86.4% (VP, Table 13) to 85.5% (VP, Table 16). It can be seen that although the accuracy is reduced, the best result is always obtained by combining all the features through the VP classifier. This also shows that this method still has a good effect on application classification. Its confusion matrix is shown in Table 17.

#### 4.2.3. Knowledge Visualization Analysis by BNOI Versus Co-Word Network

**Figure 5:**
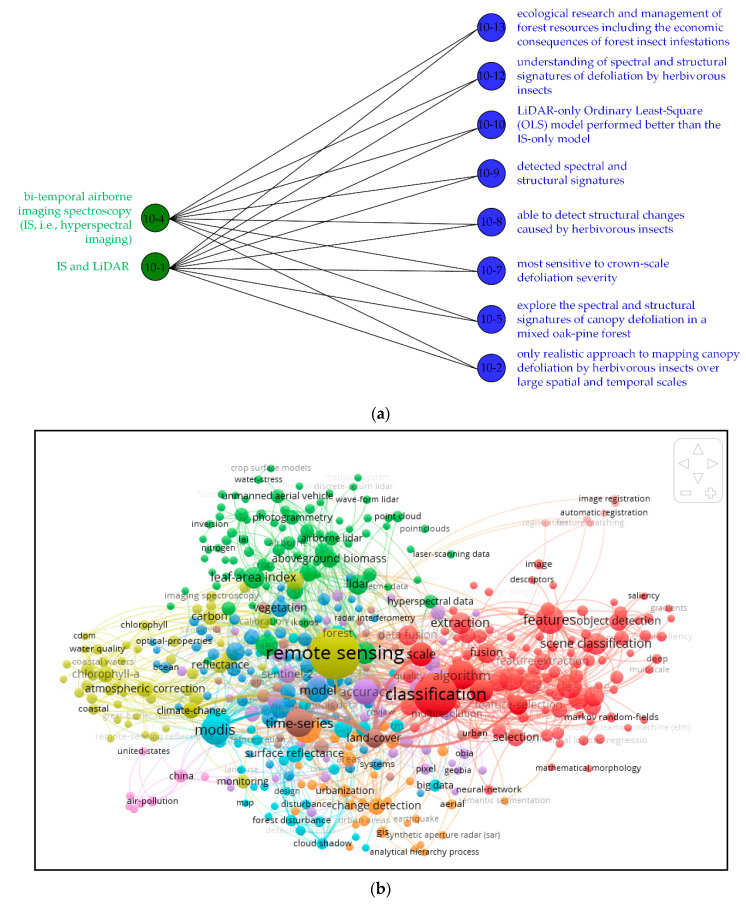
Visualization of BONI versus co-word network: (**a**) BNOI visualization of “sensors” and “applications” (the labels are the index of the sentences, “10-4” means the word comes from the fourth sentence of tenth article, green stands for the sensors and blue stands for the applications, note as the sentence is often long, we extract some representative words from the sentences. This process can also be automatic in our later future work); (**b**) Co-word network without the previous classification process visualized in VoSviewer (Sizes of the labels and nodes correspond to the frequency of the words in the papers. Different color stands for different types of the topic clusters).

Figure 5 shows the BNOI visualization of interested knowledge pair of “sensors” and “applications” and the traditional co-word network generated by VoSviewer. Note that VoSviewer is a powerful bibliometric software for visualizing the bibliometric information. Besides co-word network, other types of network like our BNOI can also be visualized by VoSviewer.

In Figure 5a, the left side is the specific sensors, and the applications corresponding to the sensors are on the right. We have counted the paired information of sensors and applications in the data set. There are about 4500 pairs of information in total, which is similar to the information we used before using the Stanford CoreNLP analysis tool. For the data of 350 articles in this experiment, approximately 12 pairs of information appear in the same article. This work is a further expansion of this experiment, readers can add knowledge quickly, conveniently, and intuitively. The classification results with highest accuracy will be the input of building a BNOI graph (that is, sensors result classified using index 1, 3, 5, 7 and applications results classified using index 1, 3, 6, 9). Also, the lemmas are used to index terms in the articles. Nowadays, technology is changing with each passing day, “sensors” and “applications” are also constantly evolving to new combinations, which are important for inspiring researchers to design their research workflow. From this figure, we can easily understand some applications of some sensors. Bipartite network is widely deployed in modeling the complex network because it brings the computational efficiency. In term of visualization readability, they also bring improvements by comparing the general network like the co-word network in Figure 5b.

Scientometric or bibliometric tools should be furtherly deployed in widely spreading and active evolving disciplines. However, there are problems for domain experts to understand the bibliometric studies for limited depth of the analysis. For domain experts, the needed knowledge is often specific and displays certain tendencies. BNOI presented in Figure 5a conveys more specific information for the bipartite connection part between “sensors” and “applications”, thus is more informative than the co-word network. The general co-word network can be improved by previous classification process in our work. We need to note that the process taken in our approach will inevitably take much more time for the previous classifications. For many scenarios, VoSviewer is enough for the displaying for the domain hotspots and suitable for newcomers in the field. Only the experts, who already know much about the field, will need the BNOI reported in our work.

### 4.3. Result Analysis and Discussion

In this work, classical classifiers (NB, SVM, and LR) and a total of nine feature extraction methods (traditional and NNLM) are employed. Then two experiments use different feature combinations and a VP method based on multi-attribute group decision-making theory. The experimental results demonstrate that our approach have provided a useful way to identify the bipartite knowledge pairs of “sensors” and “applications”.

From the perspective of feature selection, combinations of the features are efficient way to increase the classification performance. For the classification of “sensors”, the text content is mostly a proper noun and the entity boundary is clear; for the classification of “applications”, the entity boundary is not clear, and it is difficult to effectively classify it from the perspective of a single keyword. For these two classification tasks, there are some biased phrases. Therefore, we delete some biased words, and combine traditional models and NNLM for feature fusion. The results of ten-fold cross-validation show that the F-measure of “sensors” is 93.2%, and the F-measure of “applications” is 85.5%.

From the perspective of the classifier, the highest F-measure of the LR, NB, and SVM classifiers for the classification of “sensors” is 88.6%, 87.4% and 88.7%, respectively. The VP classifier obtained the highest F-measure of 93.2% in the multi-feature fusion method. For the classification of “applications”, the highest F-measure of the LR, NB, and SVM classifiers is 75.6%, 83.4% and 82.3%, respectively. The VP classifier also obtained the highest F-measure of 85.5% in the multi-feature fusion method. This shows that the VP classifier proposed in this paper can improve the classification performance.

From the perspective of comprehensive use, the main purpose of researchers to search the literature is to get more content of interest. For the classification of “sensors”, the better solution is “ {1, 3, 7, 8} + VP”(1 for Unigrams(BOW), 3 for (1-2)-grams(BOW), 7 for FastText, and 8 for ELMo), which has the highest recognition performance; for the classification of “applications”, the optimal solution is “ {1, 3, 6, 9} + VP “ (1 for Unigrams (BOW), 3 for (1-2)-grams (BOW), 6 for Doc2Vec, and 9 for BERT), which can more comprehensively identify the “applications” in the article. It can be seen from Table 11 and Table 17 of the confusion matrix that the VP classifier can obtain more positive samples, and has higher accuracy, recall rate and F-measure, which can meet the needs of researchers.

Finally, the BNOI visualization is built and compared with the co-word network. We can tell the BNOI visualization show the knowledge pair of “sensors” and “applications”, which is more specific than general co-word network visualization. Considering the domain experts, mapping of the BNOI type are surely more suitable and interesting. Thus, the approach proposed in our paper can be regarded as being superior in qualitative readability for domain experts. With the literature explosion, the readers from expert groupings should be highly emphasized, our work can thus fulfill this demand.

The BNOI also has the disadvantages because of its nature for additional classification processes. BNOI always takes more time than the traditional co-word network construction. And the BNOI mapping should not be prepared for the newcomer in the field. Because concrete knowledge entities in a BNOI may be too concrete, newcomers without enough background knowledge may feel it is hard to follow.

## 5. Conclusions

Mining the literature for finding the innovations have been proposed in TRIZ theory. With the current advanced text mining methodology, more innovation rules may be revealed. Our work used the hybrid feature extraction and classification approach to provide a bipartite network of interest (BNOI), expected to better serve researchers to identify new innovations more effectively. Through the quantitative performance evaluation and qualitative visualization, we demonstrated that our approach can help find concrete knowledge of interest to experienced researchers.

To be specific, our proposed approach extracts the bipartite knowledge pairs automatically with relatively high F-measure and the BNOI is obtained based on the classification results. The automatic process is verified by the classification F-measure obtained by the ten-fold cross-validation of the collected “remote sensing” field articles. The increase of the quantitative performance is due to the feature selection process and the classifier assembling process. Feature selection identifying features and the voting principle to assemble the classifiers are important. In this article, the comprehensive model significantly improves both tasks. In addition, biased phrases such as “remote”, “sense”, and “application” have been removed from the text. Although these features can be identified as positive samples, they are meaningless to researchers. Lastly, the classification-results based BNOI is built. The qualitative characteristics of the BNOI like readability and informative towards domain readers are also discussed by comparing with the co-word network. A classification-results based BNOI is built and demonstrated to be effective for domain knowledge understanding.

Compared with the work Heffernan and Teufel [17], they have the problems and solutions tagged, which is different from our targets of “sensors” and “applications”, so we cannot directly apply their method. In the feature extraction and classification model part, we conducted similar classification with newer methods like FastText, Bert models. Moreover, we put this one step further by applying the VP to combine different classifiers to achieve better classification results. The classification F-measure using our proposed VP classifier increases from 88.7% (sensors) and 83.4% (applications) by methods in Heffernan and Teufel [17] to 93.2% (sensors) and 85.5% (applications), respectively. Thus, our proposed method is demonstrated to be efficient.

In the future, we plan to create a larger corpus that contains more sensors and applications. In one aspect, the classification performance can be improved. The increase of amount of data may help build a better classification model. Also, both academic and industrial fields studying feature extraction and classification models. Thus, updating the classification model with the state of the art will also be necessary. In another aspect, the object of this experiment is mainly about scientific text in the remote sensing field, and applying the approach to other scientific fields will be a topic in our next work. “Sensors” and “applications” stand for the type of “data” and “applications” research, should be applied to other similar questions. The follow-up research is how to learn and applied the obtained patterns to another field. Transfer learning seems promising to help solve the problem and it is also an important research hotspot. Therefore, our next work will deal with how to transfer the knowledge of “sensors” and “applications” pairs to other similar knowledge pairs. We will explore the approach in this paper to other fields, such as extracting knowledge pairs of the “nature of proteins” and “their applications in medicine”, the “natural pests in agriculture” and “their solutions”.

## Figures and Tables

**Table 1:** Selected words and phrases for sensors.

Thermal Infrared (TIR)	Aerial	Terrestrial Laser Scanning (TLS)	Spectrum	Synthetic Aperture Radar (SAR)	Moderate Resolution Imaging Spectrometer (MODIS)
Microwave	Landsat	Imager	Satellite image	Lidar	Beidou
Sentinel					

**Table 2:** Selected words for applications.

Observation	Navigation	Mapping	Characterization	Identification
Detecting	Exploring	Photogrammetry		

**Table 3:** Confusion matrix for category C.

Ground Truth	Predicted: Text Belong to C	Predicted: Text Not Belong to C
Text belong to C	*TP*	*FN*
Text not belong to C	*FP*	*TN*

**Table 4:** Comparison of lemmatization and non-lemmatization.

Index	Feature Sets	LR			NB			SVM		
		P	R	F	P	R	F	P	R	F
1	Unigrams(BOW) ^1^	90.9	81.6	85.9	39.3	54.7	45.8	98.9	27.2	42.6
2	Unigrams(BOW) ^2^	89.5	82.5	85.9	41.1	64.7	50.2	96.3	32.2	48.2
3	Word2Vec ^1^	79.0	72.8	75.8	67.1	77.2	71.8	82.6	75.6	79.0
4	Word2Vec ^2^	77.9	74.7	76.2	64.4	78.4	70.7	81.6	75.5	78.4

^1^: No Lemmatization. ^2^: Lemmatization.

**Table 5:** The number of features using CHI.

Task	No CHI	CHI	Reduction Rate
“Sensors” or not “Sensors”	5293	879	83.4%
“Applications” or not “Applications”	5293	970	81.7%

**Table 6:** Results of classification evaluation index using LR, NB and SVM (%).

Index	Feature Sets	LR			NB			SVM		
		P	R	F	P	R	F	P	R	F
1	Unigrams (BOW)	94.3	83.4	88.5	87.3	72.7	79.3	85.5	86.1	85.8
2	Unigrams (TF-IDF)	94.2	74.0	82.8	85.4	68.7	76.1	85.6	86.2	85.8
3	(1-2)-grams (BOW)	95.4	83.9	89.2	89.6	88.4	88.9	86.7	89.3	88.0
4	(1-2)-grams (TF-IDF)	95.2	72.1	82.0	87.9	88.6	88.2	84.7	89.9	87.2
5	Word2Vec	82.5	74.2	78.1	68.7	83.5	75.3	85.8	82.9	**84.3**
6	Doc2Vec	77.0	63.7	69.7	66.5	86.0	74.9	84.5	82.7	**83.5**
7	FastText	82.8	76.2	79.3	71.5	84.9	77.5	87.9	83.5	**85.6**
8	ELMo	82.8	82.9	82.8	61.6	85.6	71.6	88.0	82.3	85.0
9	Bert	82.1	81.0	81.5	63.5	83.9	72.3	85.7	78.6	**82.0**
10	All features	89.4	87.9	88.6	89.0	88.4	88.7	85.9	92.2	88.9
11	All features {Index 1, 3, 5, 7}	93.0	86.8	89.8	89.7	88.4	89.0	85.6	91.8	88.6

**Table 7:** Results of classification evaluation index using VP (%).

Index	Feature Sets	P	R	F
1	Unigrams (BOW)	92.4	90.3	**91.3**
2	Unigrams (TF-IDF)	87.8	85.6	**86.7**
3	(1-2)-grams (BOW)	93.2	92.6	**92.9**
4	(1-2)-grams (TF-IDF)	88.8	90.9	**89.8**
5	Word2Vec	79.8	83.6	81.6
6	Doc2Vec	77.2	85.6	81.1
7	FastText	82.7	86.1	84.3
8	ELMo	85.1	86.7	**85.9**
9	Bert	80.6	83.4	**82.0**
10	All features	91.5	94.0	**92.7**
11	All features {Index 1, 3, 5, 7}	94.5	93.6	**94.0**

**Table 8:** The chi-square statistic value from the unigrams (BOW) in previous binary classification of “sensors” and “non-sensors”.

Features	Score	*p*_Value
Remote	578.28	8.86 × 10^−128^
Sense	391.56	3.78 × 10^−87^
Spectral	246.88	1.24 × 10^−55^
Sensing	236.08	2.81 × 10^−53^
Resolution	198.31	4.87 × 10^−45^
Image	164.17	1.39 × 10^−37^
Modis	150.89	1.11 × 10^−34^
Hyperspectral	149.00	2.86 × 10^−34^
Landsat	143.34	4.94 × 10^−33^
Radar	143.34	4.94 × 10^−33^

**Table 9:** Results of classification evaluation index using LR, NB and SVM (%) (bias words like “remote” and “sense” removed).

Index	Feature Sets	LR			NB			SVM		
		P	R	F	P	R	F	P	R	F
1	Unigrams (BOW)	90.5	76.2	82.7	87.2	71.9	78.7	83.6	79.5	81.4
2	Unigrams (TF-IDF)	90.2	68.2	77.6	85.3	68.1	75.6	83.6	79.5	81.5
3	(1-2)-grams (BOW)	92.8	77.0	84.1	89.2	85.6	87.4	85.5	84.4	84.9
4	(1-2)-grams (TF-IDF)	90.5	66.8	76.8	87.6	85.7	86.6	83.1	85.2	84.1
5	Word2Vec	76.3	68.2	71.9	63.8	82.8	72.0	81.8	76.2	**78.9**
6	Doc2Vec	75.7	60.1	67.0	62.5	84.6	71.8	81.8	77.8	**79.7**
7	FastText	77.8	69.5	73.4	64.4	84.0	72.9	83.1	77.2	80.0
8	ELMo	78.0	81.3	79.6	61.3	84.2	70.9	83.6	78.7	81.1
9	Bert	75.3	73.9	74.5	60.5	79.8	68.8	82.0	70.6	75.8
10	All features	89.1	87.6	88.3	88.7	85.6	87.1	85.3	90.4	87.7
11	All features {Index 1, 3, 7, 8}	90.0	87.2	88.6	89.4	85.6	87.4	86.6	91.0	88.7

**Table 10:** Results of classification evaluation index using VP (%) (bias words like “remote” and “sense” removed).

Index	Feature Sets	P	R	F
1	Unigrams (BOW)	90.0	80.3	**86.0**
2	Unigrams (TF-IDF)	86.6	78.0	**82.0**
3	(1-2)-grams (BOW)	94.6	87.1	**89.3**
4	(1-2)-grams (TF-IDF)	88.2	86.8	**87.4**
5	Word2Vec	74.9	80.9	77.8
6	Doc2Vec	72.5	83.5	77.6
7	FastText	79.8	83.3	**81.5**
8	ELMo	81.8	82.8	**82.3**
9	Bert	75.6	79.0	**77.2**
10	All features	90.8	92.7	**91.7**
11	All features {Index 1, 3, 7, 8}	93.1	93.3	**93.2**

**Table 11:** Confusion matrix for sensors in remote sensing.

Ground Truth	Predicted: Sensors	Predicted: Not Sensors
Sensors	107	8
Not sensors	8	209

**Table 12:** Results distinguishing applications from non-applications using LR, NB, SVM.

Index	Feature Sets	LR			NB			SVM		
		P	R	F	P	R	F	P	R	F
1	Unigrams (BOW)	81.4	69.0	74.6	81.7	56.2	66.5	76.4	73.3	74.8
2	Unigrams (TF-IDF)	78.8	59.3	67.6	76.8	49.2	59.9	75.3	70.5	**72.7**
3	(1-2)-grams (BOW)	84.5	69.1	75.9	86.8	80.8	83.6	84.4	81.4	82.9
4	(1-2)-grams (TF-IDF)	78.4	55.6	64.9	85.2	80.8	82.9	80.9	81.6	81.2
5	Word2Vec	67.4	57.0	61.7	61.6	80.8	69.9	76.7	70.2	**73.2**
6	Doc2Vec	66.9	50.7	57.6	63.0	82.0	71.2	76.9	72.0	**74.3**
7	FastText	66.9	54.3	60.0	60.0	80.2	68.6	76.7	68.6	**72.4**
8	ELMo	38.9	67.6	68.2	55.9	81.7	66.3	78.6	70.9	**74.5**
9	Bert	71.7	68.7	70.1	60.9	80.2	69.2	77.4	69.5	73.2
10	All features	75.5	73.3	74.3	86.0	80.7	83.2	80.6	84.8	82.6
11	All features {Index 1, 3,6, 9}	79.1	72.2	75.4	76.8	80.6	83.5	83.0	83.0	82.9

**Table 13:** Results of classification evaluation index using VP (%).

Index	Feature Sets	P	R	F
1	Unigrams (BOW)	83.8	71.6	**77.2**
2	Unigrams (TF-IDF)	78.8	62.1	69.4
3	(1-2)-grams (BOW)	88.0	82.5	**85.1**
4	(1-2)-grams (TF-IDF)	85.2	80.9	**83.0**
5	Word2Vec	66.9	77.6	71.8
6	Doc2Vec	67.8	80.2	73.4
7	FastText	67.8	77.3	72.2
8	ELMo	71.1	76.3	73.6
9	Bert	70.5	76.9	**73.5**
10	All features	86.0	84.0	**84.9**
11	All features {Index 1,3,6, 9}	88.8	84.2	**86.4**

**Table 14:** The chi-square statistic value from the (1-2)-grams (BOW) in previous binary classification of “applications” and “non-applications”.

Features	Score	*p*_Value
Satellite	191.98	1.17 × 10^−43^
Remote	168.35	1.69 × 10^−38^
Monitoring	140.93	1.67 × 10^−32^
Resolution	140.74	1.84 × 10^−32^
Sense	109.07	1.45 × 10^−25^
Remote sense	86.26	1.58 × 10^−20^
Observation	85.95	1.85 × 10^−20^
Modis	84.80	3.31 × 10^−20^
Lidar	82.12	1.28 × 10^−19^
Remote sensing	80.69	2.64 × 10^−19^

**Table 15:** Results distinguishing applications from non-applications using LR, NB, SVM (bias words like “application” removed).

Index	Feature Sets	LR			NB			SVM		
		P	R	F	P	R	F	P	R	F
1	Unigrams (BOW)	80.9	68.6	74.1	81.7	56.2	66.4	76.1	72.2	73.8
2	Unigrams (TF-IDF)	79.3	57.6	66.6	76.5	49.0	59.7	74.8	68.6	**71.5**
3	(1-2)-grams (BOW)	84.3	68.7	75.6	86.9	80.4	83.4	83.8	80.5	82.0
4	(1-2)-grams (TF-IDF)	78.6	53.8	63.8	85.3	80.5	**82.7**	80.8	81.4	81.0
5	Word2Vec	65.4	54.3	59.2	58.0	81.3	67.7	76.0	70.3	**73.0**
6	Doc2Vec	65.0	48.0	55.2	61.4	82.8	70.5	75.5	73.1	**74.2**
7	FastText	65.5	53.7	58.9	56.8	80.1	66.4	75.4	59.3	**72.2**
8	ELMo	67.9	64.6	66.2	56.3	80.5	66.3	77.5	68.7	72.8
9	Bert	70.5	68.4	69.4	61.0	80.4	69.3	77.6	69.4	73.2
10	All features	75.5	73.6	74.5	86.0	80.3	83.0	80.8	84.1	82.3
11	All features {Index 1, 3,6, 9}	78.0	71.4	74.5	86.9	80.2	83.4	82.9	81.6	82.2

**Table 16:** Results of classification evaluation index using VP (%) (bias words like “application” removed).

Index	Feature Sets	P	R	F
1	Unigrams (BOW)	83.5	70.9	**76.7**
2	Unigrams (TF-IDF)	78.4	61.3	68.8
3	(1-2)-grams (BOW)	87.7	82.1	**84.7**
4	(1-2)-grams (TF-IDF)	85.4	80.4	**82.8**
5	Word2Vec	66.6	77.8	71.8
6	Doc2Vec	66.6	80.8	73.0
7	FastText	67.0	76.9	**71.5**
8	ELMo	69.7	74.6	**72.1**
9	Bert	70.4	77.0	**73.5**
10	All features	86.1	83.1	**84.5**
11	All features {Index 1,3,6, 9}	88.1	83.2	**85.5**

**Table 17:** Confusion matrix for VP classifier.

Ground Truth	Predicted: Application	Predicted: Not Application
Application	96	20
Not application	13	203

## Data Availability

The classification datasets are uploaded to the github site. It is open access for readers. https://github.com/15514783351/RemoteSensing Available from 30 October 2020.
